# Responding to COVID-19 threats to trial conduct: lessons learned from a feasibility trial of a psychological intervention for South African adolescents

**DOI:** 10.1186/s13063-021-05400-8

**Published:** 2021-07-09

**Authors:** Bronwyn Myers, Claire van der Westhuizen, Megan Pool, Nancy Hornsby, Katherine R. Sorsdahl

**Affiliations:** 1 Alcohol, Tobacco and Other Drug Research Unit, South African Medical Research Council, PO Box 19070, Tygerberg, 7505 South Africa; 2 Curtin enAble Institute, Faculty of Health Sciences, Curtin University, Perth, WA Australia; 3 Department of Psychiatry & Mental Health, University of Cape Town, Cape Town, South Africa; 4 Alan J Flisher Centre for Public Mental Health, Department of Psychiatry & Mental Health, University of Cape Town, Cape Town, South Africa

**Keywords:** COVID-19, Adolescent, Mental health, Low- and middle-income country, Quality improvement cycle

## Abstract

**Abstract:**

The COVID-19 pandemic has posed challenges to the conduct of clinical trials. Strategies for overcoming common challenges to non-COVID-19 trial continuation have been reported, but this literature is limited to pharmacological intervention trials from high-income settings. The purpose of this paper is to expand the literature to include a low- and middle-income country perspective. We describe the challenges posed by COVID-19 for a randomised feasibility trial of a psychological intervention for adolescents in Cape Town, South Africa, and lessons learned when implementing strategies to facilitate trial continuation in this context. We used a Plan-Do-Study-Act cycle method to explore whether our adaptations were having the desired effect on trial accrual and retention. We found that stakeholder engagement, trial coordination and team communication need to be intensified while testing these procedural changes. We learned that strategies found to be effective in high-income countries required significant adaptation to our resource-constrained setting. The detailed documentation of extraneous influences, procedural changes and trial process information was essential to guiding decisions about which adaptations to retain. This information will be used to examine the potential impact of these changes on study outcomes. We hope that these reflections will be helpful to other trialists from low- and middle-income countries grappling with how to minimise the impact of public health emergencies on their research.

**Trial registration:**

The trial is registered with the Pan African Clinical Trials Registry (PACTR20200352214510). Registered 28 February 2020.  https://pactr.samrc.ac.za/TrialDisplay.aspx?TrialID=9795.

## Background

Despite the promise of a vaccine, the COVID-19 pandemic continues to pose a threat to public health [1]. In response to this global emergency, countries have implemented various measures to contain the spread of the disease and preserve health system capacity by promoting social distancing and restricting freedom of movement [2].

While necessary, these restrictions have had a profound effect on non-COVID-19 clinical research. Around the world, non-COVID-19 clinical trials have been suspended or halted due to COVID-19 regulations, sponsor or funder directives and operational challenges [3–5]. Non-COVID-19 trials permitted to continue or resume and required substantial adjustments to recruitment, intervention, data collection and trial management protocols in response to the social distancing and quarantine challenges posed by COVID-19 [6, 7]. Recent systematic reviews and studies have reported that these mitigation efforts have resulted in enrolment delays and operational gaps that have negatively impacted on trial timelines and outcomes [8, 9].

Various regulatory authorities have issued guidance for trial conduct during the pandemic that includes recommendations for adaptations to ensure participant safety [10–13]. This guidance recommends virtual recruitment and informed consent processes; the off-site administration of investigational products and procedures for virtual data collection, remote study and safety monitoring, remote data management; and the implications of these adaptations for statistical analysis [10–14]. While some of the guidance (e.g. [12]) details procedures for obtaining informed consent virtually, the off-site provision of investigational products and remote data management, for the most part available guidance, offers few specifics on how to adapt trial procedures to facilitate continuation while minimising risks to trial integrity [14, 15]. Additionally, this guidance is largely focused on trials of pharmacological interventions and does not address issues around the remote delivery and fidelity monitoring of non-pharmacological interventions.

To help operationalise this guidance, trialists are sharing their strategies for mitigating against COVID-19 challenges to trial conduct. These challenges include restricted access to health services, difficulty in recruiting participants due to stay-at-home orders, concern that trial procedures increase the risk of COVID-19 exposure for participants and staff, higher attrition rates due to difficulties in contacting participants and logistical challenges related to access to personal protective equipment (PPE) [6, 7, 14–18]. While helpful, the literature remains largely limited to trials conducted in high-income countries (HICs). This is not surprising given the underrepresentation of low- and middle-income countries (LMICs) in trials methodology research [19]. However, the growing investment in global health trials [20] makes this an important gap to address. Although many of the challenges described by trialists in HICs are relevant, they are likely to be exacerbated in trials conducted in LMICs due to pre-existing logistical, resourcing and implementation issues that make it difficult to conduct trials in these settings [21–23]. Compared to HICs, trialists in LMICs have substantially less access to health facilities and other infrastructure required to conduct trials, trained human resources and methodological expertise, and financial resources to support clinical trials [21, 22]. Additionally, COVID-19 restrictions may amplify the context-specific challenges that affect participant enrolment and retention in trials within LMICs [22]. These include limited patient education and support for trial participation; widespread socio-economic adversity that limits access to technology, transport and time to participate in trials; and greater participant mobility (due to unstable housing and migrant labour). As a result of these contextual, systemic and population differences between HICs and LMICs, strategies for mitigating COVID-19 obstacles to trial conduct in HICs (such as online recruitment and data collection) may be less feasible to use in LMICs.

The purpose of this paper is to expand the discussion on trial continuation during COVID-19 to include a LMIC perspective. We describe the challenges posed by COVID-19 for an ongoing randomised feasibility trial of a psychological intervention for adolescents in Cape Town, South Africa, and the strategies employed to facilitate trial continuation. We hope that these reflections will be helpful to other trialists from LMICs grappling with how to minimise the impact of public health emergencies on their research.

## Trial description

Recruitment for a randomised feasibility trial of an intervention for reducing risk for depression and heavy alcohol use among South African adolescents (aged 15–18) began on 04 November 2019. The original protocol is described in [24] (trial registration: PACTR20200352214510). Prior to COVID-19, field staff recruited adolescents from disadvantaged neighbourhoods using community-based outreach techniques. These involved approaching young people in places that adolescents frequent to describe the study and conduct eligibility screening after obtaining their verbal consent. The staff met with the parents of eligible adolescents younger than 18 years old to describe the study and obtain written informed consent for their child’s participation. After obtaining parental consent, the adolescent was invited to a study enrolment visit. At this first appointment, their written informed assent for study participation was obtained, an interviewer-administered baseline assessment conducted and the first counselling session was delivered before randomly assigning participants to the ASPIRE intervention (three additional counselling sessions and referral to other services) or a comparison condition (referral to other services). Counselling was supported via a handbook summarising the content of each session and containing worksheets for practising the problem-solving method. All participants were to be physically tracked in their communities to facilitate scheduling for the 6-week and 3-month post-randomisation assessments where the baseline questionnaire was re-administered. All activities occurred at a dedicated clinical research site.

## The COVID-19 trajectory in South Africa

**Fig. 1:**
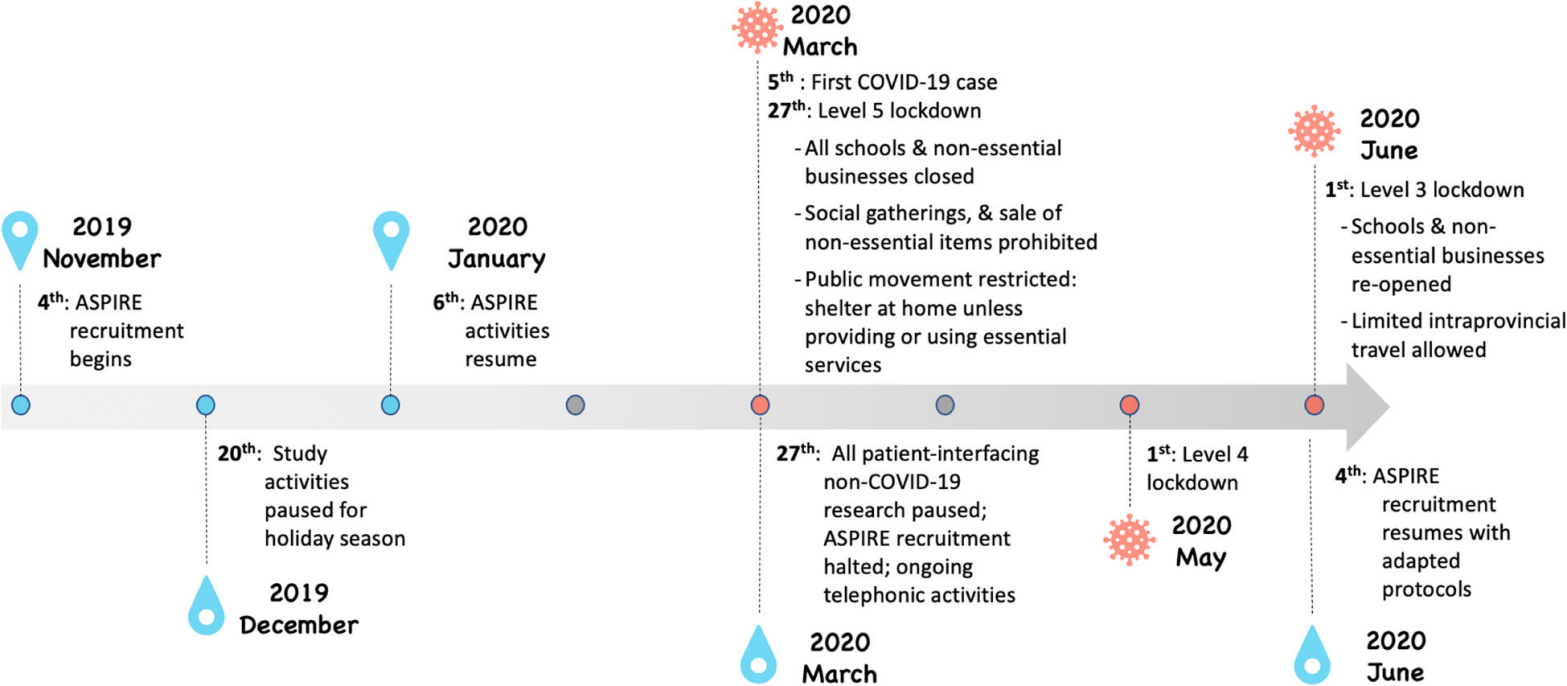
Timeline of the ASPIRE trial and COVID-19 pandemic in South Africa

On 27 March 2020, South Africa entered a strict national “level 5” lockdown that involved strict restrictions to limit the spread of the virus (see Fig. 1), including the prohibition of the sale and distribution of alcohol [25]. This led to all patient-interfacing non-COVID-19 research being paused. By this time, the ASPIRE trial had enrolled 67 participants of a planned 100. When research was paused, only 15 of the 67 had completed the full protocol. Although we were permitted to use remote procedures to complete the study activities for participants enrolled in the trial, all new recruitment was suspended. These restrictions were gradually lifted as the country moved through the first wave of infections, with some ethics committees permitting non-COVID-19 trials to resume recruitment under strict conditions during level 3 lockdown [26].

## Sponsor and ethics committee stipulations for full trial resumption

Prior to requesting resumption of recruitment, the principal investigators (PIs) engaged with the sponsor (South African Medical Research Council [SAMRC]) and the two South African ethics committees that approved the original trial (SAMRC, University of Cape Town (UCT)) to understand their requirements and conditions. The trial sponsor and the SAMRC human research ethics committee were willing to permit full trial resumption, provided that field staff were trained in and implemented COVID-19 safety protocols, all staff and participants completed daily COVID-19 screening and were provided with PPE, and activities occurred in spaces that allowed for good ventilation and social distancing. However, the team was encouraged to limit face-to-face participant contact. UCT’s Faculty of Health Sciences’ Human Research Ethics Committee had stricter requirements, only permitting remote contact between UCT-employed field staff and participants. To ensure parity and risk mitigation for all staff, the PIs substantially adapted the trial protocols to minimise in-person contact.

## Adaptations to trial procedures in response to COVID-19

On 4 June 2020, the ASPIRE trial was granted ethical approval to resume recruitment with adapted protocols (described below). Although some of these adaptations posed potential challenges to the feasibility outcomes and quality of the trial, they also presented opportunities to test alternative ways of conducting mental health intervention trials in resource-constrained settings.

### Methods used to guide decisions about trial adaptations

We used the Plan-Do-Study-Act (PDSA) method [27] to guide decision-making about the required protocol changes. This approach was selected as it is pragmatic, facilitates rapid assessment of small changes to current procedures and uses feedback from the assessment to improve the fit of the solution for the context [27, 28]. This method involves a four-stage cycle: the “plan” stage involves identifying the desired change, the “do” stage involves implementing the change, the success of this change is evaluated in the “study” stage and further refinements to the original change are identified in the “act” stage which informs the next cycle of practice improvement [28, 29].

First, the investigators mapped out the potential obstacles to trial recruitment, data collection and intervention delivery and discussed possible strategies for addressing these barriers during video calls with the funder representative, sponsor, the institutional ethics committees and the trial steering committee. Feedback from these meetings guided the construction of a menu of possible protocol change options. We amended our protocol and sought ethics approval for the proposed strategies for facilitating trial continuation. We submitted a single request for a protocol amendment that described each of the strategies and how we would implement these. Following approval, we incrementally implemented the proposed changes to recruitment, data collection and intervention delivery procedures. During weekly video calls with the trial and counselling coordinators, the investigators used trial process data (accrual rate, attrition, intervention sessions completed) to review and refine the recruitment, data collection and intervention protocols. The recruiters and data collectors met weekly with the trial coordinator to provide feedback on how well the changes had worked and ways in which these could be refined. The counsellors provided similar feedback to the counselling coordinator during their weekly meetings. The coordinators shared the minutes of these meetings and verbal feedback with the investigator team. This was also used to inform further refinements to trial procedures as needed.

### Changes to recruitment and consent procedures

In response to COVID-19 concerns, we paused community screening and recruitment, switching to a fully remote process where adolescents could self-refer for eligibility screening via telephone. To facilitate accrual, we marketed the study by distributing study flyers and posters at places accessible to young people during the pandemic, on social media and to organisations providing COVID-19 relief services. Marketing materials contained a telephone number that adolescents could contact for further information. We also adapted parental consent and adolescent assent/consent procedures so that consent/assent could be obtained via telephone, with verbal consent being audio-recorded for audit purposes. After participants were enrolled in the study, the field team delivered hard copies of participant information leaflets and consent/assent forms to the participant and collected signed copies of these forms.

Although necessary, these changes affected the trial’s accrual rate, with more than a 50% reduction in monthly screening totals observed after switching to telephone screening. This is not surprising as active case finding through outreach is considered more effective than self-referral for recruiting adolescents into psychological interventions [30, 31]. With these changes, we observed an increase in potential participants lost between screening and informed consent/assent procedures. The proportion of eligible adolescents whom we were unable to locate for informed consent/assent increased from 8.7 to 15.6% (*p* < 0.001) and the proportion of parents from whom we were unable to obtain parental consent for their child’s participation increased from 8.7 to 17.9% (*p* < 0.001).

The switch to remote recruitment likely affected recruiters’ ability to build rapport and gain the trust of potential participants and their parents. Prior to COVID-19, we had purposefully employed recruiters known and respected in the communities to address issues of community mistrust of unknown researchers, a well-documented barrier to adolescent participation in clinical research [31]. Their good reputation in the community and “references” from other community members often served as an entry point with adolescents and parents, affording recruiters the opportunity to meet with parents to discuss the study and allay any concerns related to their child’s participation. With the remote screening and consent processes, parental consent was more challenging to obtain with several being hesitant or refusing to allow their child to participate in a study with strangers.

To address this obstacle, we asked trusted community leaders and organisations providing support services to vulnerable children and families to vouch for our trustworthiness and refer adolescents who they think have unmet psychosocial needs to ASPIRE for screening. The personal recommendations of these individuals and organisations appear to have improved the willingness of parents and adolescents to engage with the ASPIRE team.

### Changes to data collection procedures

In addition, we changed our protocols to facilitate telephonic data collection. While expedient, there were logistic and environmental barriers to using this approach that may have affected data quality and participant retention. Most ASPIRE participants had limited access to a telephone—they lived in overcrowded, low-income households that shared a single phone. These living circumstances were not conducive to privacy, potentially impacting their willingness to disclose sensitive information via telephone. With telephonic data collection, field staff missed out on important non-verbal clues for when to probe participant responses or rephrase sensitive questions for enhanced accuracy. The use of video call platforms like Zoom and Skype may have overcome this challenge, but most adolescents in our study had limited access to the hardware needed to support this technology. Poor connectivity (with calls disconnecting or being of poor quality) and background noise were other issues that impacted telephonic data collection. We explored whether providing airtime or data to adolescents improved their participation in the trial but soon abandoned this strategy when it became clear that hardware access and privacy concerns were the primary barriers to telephonic data collection.

To limit the impact of these challenges on data quality, we trained staff to adopt a flexible approach to scheduling telephonic assessments at a time when participants had access to a telephone and sufficient privacy. Second, to minimise the impact of participant fatigue and call interruptions on data quality, we re-ordered the questionnaires to prioritise the collection of primary outcome data before asking about secondary outcomes. Third, we obtained ethical approval to audio-record assessments to monitor data collection quality. We have used these recordings to verify the data captured on paper case report forms and as a tool for training data collectors in strategies for enhancing data quality.

Despite these efforts, we suspect that switching to telephonic data collection contributed to a higher than the anticipated loss to follow-up at the 6-week and 3-month endpoints (20.5% at 6-week follow-up and 21.4% of participants scheduled for a 3-month assessment). Notably, 87.5% of the participants lost for the 6-week follow-up, and 91.3% of participants lost at the final endpoint occurred when we were unable to physically track participants to remind them of their appointments. Telephonic assessments may have been a less engaging experience for adolescents than face-to-face assessments at our research site—a place designed to be a welcoming environment for young people.

### Changes to intervention protocols

The intervention was also adapted for telephone delivery. The shift from face-to-face to tele-counselling affected the therapeutic alliance between counsellors and participants, particularly for newly enrolled participants who seemed less likely to engage and complete the intervention if the initial session was delivered via telephone. Specifically, 95.0% of participants who received face-to-face counselling in session one completed session 2 compared to 84.2% of participants who obtained session 1 via telephone. In addition, 56.1% of participants who received face-to-face counselling in session 1 completed all four counselling sessions compared to 42.1% of participants whose initial exposure to the counselling intervention occurred via telephone.

Counsellors reported greater difficulty in teaching behavioural skills via telephone without being able to gauge participants’ comprehension of counselling content. The logistic and environmental barriers to telephonic data collection, described earlier, also impacted counselling. Call interruptions impacted the flow and duration of sessions. The lack of privacy to openly discuss problems was a concern for several participants, raising questions about their ability to fully engage in tele-counselling. COVID-19 also affected the team’s ability to refer adolescents for additional counselling, which was key to ensuring the safety of participants with unmet psychological needs. COVID-19 restrictions also affected the community and health services to which we had previously referred adolescents. Already in scarce supply [32], these services were either non-operational or could only provide limited services via remote platforms.

To address these challenges, we introduced alternative strategies for establishing rapport, promoting counselling engagement, and ensuring participant safety. To combat participant fatigue and attention difficulties, we split the enrolment appointment into one contact for the baseline assessment and a subsequent contact for the first counselling session. Counsellors noted that this improved participants’ ability to remain engaged throughout the session. However, it also introduced additional opportunities for attrition, with seven participants being lost to follow-up before meeting their counsellor and completing any counselling sessions. Counsellors tried to limit attrition and establish initial rapport by engaging with participants through introductory pamphlets and videos to introduce themselves, remind them of their upcoming counselling appointment and educate them on what to expect from counselling.

Other strategies for reducing participant fatigue and improving attention included giving participant’s breaks to stretch their legs, introducing physical movement into the session to raise energy and limiting tele-counselling to key concepts with participants being referred to the handbook for additional content, examples and practice activities. We also delivered counselling support packages to the homes of participants randomised to the ASPIRE intervention. These contained the counselling handbooks, earphones so that the session content could be listened to without being overheard by others and a list of additional resources that a participant could access for further support. Strategies to ensure participant safety in the absence of referral agencies included additional community case management for participants identified as being at high risk for adverse mental health outcomes. Case management involved re-screening participants for CMD symptoms to assess safety risk, crisis support (if required), provision of further referrals to tele-counselling services and ongoing monitoring.

With environmental constraints continuing to impact counselling engagement for some participants, we adopted a hybrid approach to intervention delivery from November 2020 after the further easing of lockdown restrictions. Most participants still received tele-counselling, unless they expressed a preference for face-to-face counselling or counsellors perceived an elevated risk of attrition. Face-to-face counselling necessitated adjusting trial operational procedures to further mitigate COVID-19 risk for staff and participants.

### Changes to trial management and operational procedures

In addition to augmenting operational procedures to include COVID-19 safety protocols, we also adjusted team organisation, communication and staff management procedures to allow for flexibility and agility in responding to a rapidly changing landscape. To ensure study continuity in the event of a staff member becoming ill or exposed to COVID-19, we created two separate field teams to minimise the risk of the whole team being exposed to COVID-19. However, COVID-19 infections within these teams still impacted the pace of study activities. We also adjusted work schedules to allow for greater flexibility. This flexibility included working after hours and on weekends to accommodate the changing needs of participants and staff whose caregiving responsibilities increased because of school closures.

These changes increased the need for greater frequency of field team communication and intensity of team coordination. In response, we introduced daily team meetings using online platforms, a WhatsApp communication group for counsellors and recruiters to coordinate the scheduling of assessment and counselling appointments, and daily feedback from the counsellors to the counsellor supervisor to facilitate coordination of counselling activities. Training and supervision for the data collectors also moved online. Counselling supervision took place via telephone, with the supervisor available via online platforms for real-time support to address any potential challenges as staff adjusted to these new protocols. This daily communication provided team members with opportunities to share the frustrations of conducting a trial in the context of a pandemic; rapidly review and problem solve challenges to recruitment, data collection and counselling; and support team members struggling with pandemic-associated distress.

## Responding to the potential impact of COVID-19 and protocol changes on trial outcomes

Despite our efforts, the stressors and strains associated with the pandemic may have diluted the effects of the ASPIRE intervention and standard of care. The illness and death of family members, social isolation associated with lockdown regulations, school closures and loss of education time, and worsening food insecurity (due to growing unemployment and halting of school feeding schemes) have almost certainly impacted the psychological well-being of South African adolescents [33]. ASPIRE participants in the control arm received a single intervention session and referral to standard care, namely non-government organisations and public health facilities serving adolescents. With major disruptions to standard care being reported [34], difficulties in accessing standard care may inflate the effects of the ASPIRE intervention. As we did not collect detailed information on the provision of standard care, we cannot assess the impact of COVID-19 on access to standard care for ASPIRE participants. We plan to remedy this oversight in a future effectiveness trial.

For the ASPIRE trial, the country’s intermittent bans on the sale and distribution of alcohol will add another layer of complexity to the interpretation of trial outcomes related to alcohol use. These bans spanned from 27 March to 1 June 2020, 13 July to 16 August 2020, and 28 December 2020 to 1 February 2021. As adolescent alcohol use is greatly influenced by community availability [35, 36], we anticipate that these bans will impact the frequency and quantity of alcohol consumed by participants.

To account for the impact of these contextual changes on our feasibility outcomes, we are documenting government restrictions on movement, school access, alcohol, and tobacco use, and any other major disruptive events so these can be factored into the analyses and considered when interpreting results. Second, we are collecting additional information on participants’ exposure to COVID-19 and the impact of COVID-19 on access to alcohol, school attendance and learning, and mental health at each study assessment. These data will add to the emerging literature on the psychological effects of COVID-19 and lockdown restrictions on adolescents living in LMICs. Third, we are documenting the protocols under which participants are enrolled, data collected and interventions provided so that we can explore differences in feasibility (accrual, intervention and study retention rates) and clinical outcomes among participants enrolled under different protocols. While unplanned, these protocol changes will allow us to explore the feasibility of various recruitment, data collection and intervention delivery approaches that may be useful as we plan for a larger trial. To ensure we have enough participants enrolled under each protocol to allow for meaningful comparisons, we increased our recruitment target and extended our enrolment period.

## Impact of COVID-19 protocol changes on timeline and budget

Research pauses and procedural changes due to COVID-19 regulations resulted in trial recruitment taking 8 months longer than planned. This has had a knock-on effect, delaying completion of the intervention, 6-week and 3-month post-enrolment assessments, and qualitative process evaluation interviews. The trial is ongoing, with some 3-month assessments and process evaluation interviews still pending. We anticipate that the combination of these study pauses and protocol changes will extend the trial timeline by 10 months. These delays also increased the cost of conducting the trial. The original budget for field personnel (project coordinator, assessors, counsellors) increased by 48%. Although some of the protocol changes did result in cost savings (for example, the budget for travel decreased by 60%), there were other unplanned costs including computer hardware for staff, data costs and costs of PPE. Our institutions have supported us in managing these budgetary challenges by allowing investigators to charge less to the project and provide in-kind support. Since 2018, when the grant was awarded, the local currency (South African Rand) has depreciated significantly against the British pound. We have used these foreign exchange savings to counter the budgetary challenges, but the trial did not have sufficient funding to introduce other COVID-19 mitigation strategies such as rapid diagnostic testing (RDT) when these were approved for use by the South African Health Products Regulatory Authority on 25 August 2020.

## Discussion

Prior research has reported various strategies to ensure the continuation and/or resumption of non-COVID-19 trials during the pandemic [6, 7]. As these reports have been restricted in scope to pharmacological interventions and HIC perspectives, they may have limited utility to trialists testing psychological interventions in low-resource settings where trial conduct is substantially more challenging [21, 22]. This paper begins to address this gap by describing strategies employed to enable the continuation of a non-pharmacological intervention trial for adolescent populations in a LMIC during this global emergency.

Through adapting the ASPIRE trial recruitment, intervention and data collection protocols for COVID-19 responsiveness, we learned three key lessons that may be helpful to other trialists from LMICs. First, switching to remote data collection and intervention delivery is not simple to implement in low-resource settings—even though much of the COVID-19 trial guidance and literature from HICs recommends this as a mitigation strategy [10–12, 15]. Although there are concerns that remote trial processes may lead to digital exclusion of vulnerable individuals in HICs (such as individuals of low SES who face financial and privacy barriers to engagement in remote research) [37, 38], the likelihood of digital exclusion is greater in LMICs. Most of the offline population live in African LMICs [39], where much of the populace lives below the breadline and where electricity, internet and mobile telephone access is often unstable [40]. From our experience, access to mobile devices, connectivity issues (quality and interruptions) and lack of privacy within households are likely to pose significant challenges to data quality and engagement in non-pharmacological interventions in LMICs. Our team has learned the importance of acknowledging limitations to remote processes and considering tactics to minimise the impact of these limitations on trial accrual, data quality and attrition when planning for remote trial processes. The saliency of these difficulties for adolescents in the ASPIRE trial also cautions against solely relying on tele-health for mental health care provision in future trials with adolescents in LMICs [41, 42]. To avoid digital exclusion and the exacerbation of inequalities in access to services, some adolescents will continue to require face-to-face contact. As COVID-19 rapid diagnostic tests become more accessible, future trials could consider the use of these tests to further mitigate the risks of COVID-19 during face-to-face recruitment and data collection procedures.

Second, we learned the value of collecting detailed information on major disruptive events and contextual changes that impact study participants and their families. We extracted information on the nature, onset and duration of these disruptions from print documents (such as the media and COVID-19 policy documents). We also collected this information directly from study participants to enhance understanding of the personal impact of these changes. With this information, we plan to identify participant sub-groups providing outcomes during these disruptions and explore the potential impact of these changes on study outcomes. Beyond the COVID-19 pandemic, we argue that collecting detailed information on social and environmental disruptions is critical for successful trial management and should be routinely collected as part of trial monitoring, particularly in contexts where disruptions due to political conflict, community violence and other humanitarian crises commonly occur.

Third, continuous monitoring of trial process indicators is necessary for assessing whether adjustments to recruitment, data collection and intervention protocols had the desired effect. With COVID-19 being unprecedented, we lacked evidence to guide initial protocol adaptation decisions and were forced to learn by doing. Learning was facilitated through PDSA cycles. Although PDSA is a frequently used process for quality improvement in health services [27–29], it has rarely used to identify and test improvements to trial procedures.

Several lessons were learned while using PDSA to test small changes to trial procedures that may be useful to other trialists seeking to improve their procedures. Early and regular engagement with the sponsor, ethics committees and trial steering committee can assist in mapping out a menu of possible change options. We sought approval for each of these changes in a single protocol amendment, avoiding the time delays associated with multiple requests for protocol amendments. Second, implementing a series of small changes augmented the need for communication and coordination within the field team and between the field team and investigators. Field team feedback on whether changes were having the desired effect or unanticipated challenges was integral to reviewing and refining procedural adaptations. Third, the success of a PDSA cycle is contingent on collecting detailed information from trial process indicators, so the feasibility, impact on outcomes and cost of alternative recruitment, data collection and intervention delivery methods can be explored. For the ASPIRE trial, these included the (1) nature, dates and duration of all procedural changes; (2) the participants affected by these changed protocols; and (3) staff hours spent on each study activity.

To conclude, COVID-19 has forced us (like many trialists) to change our original study protocols to allow for trial continuation during this public health emergency. Unlike an effectiveness trial where numerous changes to the trial protocol would raise questions about trial integrity, this trial’s focus on feasibility rather than clinical outcomes allowed us to adapt and test a variety of study procedures [43]. These changes have presented us with an opportunity to explore the feasibility of alternative recruitment, data collection and intervention delivery methods and to gather data on how particular subgroups of participants respond to each of these methods. As part of the trial’s process evaluation, we are conducting post-study interviews with trial participants in which we will explore their experiences of the procedural changes and preferences. These data, together with the trial process data, will be used to improve the feasibility and acceptability of trial procedures employed in a definitive effectiveness trial. Further, our application of a quality improvement approach to assessing whether these alternatives improved performance on feasibility indicators may be useful to other trialists grappling with these decisions. Through responding to these threats to study completion, we have learned that stakeholder engagement, trial coordination with regular and effective team communication, and detailed documentation of extraneous influences, procedural changes and trial process information intensify in importance and are critical to evidence-based and agile responses to crises.

## Data Availability

Not applicable.

## Abbreviations

CMD: Common mental disorders
HICs: High-income countries
LMICs: Low- and middle-income countries
PI: Principal investigator
PPE: Personal protective equipment
